# A Proteomic Approach for the Identification of Up-Regulated Proteins Involved in the Metabolic Process of the Leiomyoma

**DOI:** 10.3390/ijms17040540

**Published:** 2016-04-09

**Authors:** Blendi Ura, Federica Scrimin, Giorgio Arrigoni, Cinzia Franchin, Lorenzo Monasta, Giuseppe Ricci

**Affiliations:** 1 Institute for Maternal and Child Health—IRCCS “Burlo Garofolo”, Trieste 34137, Italy; blendi.ura2006@libero.it (B.U.); federica.scrimin@burlo.trieste.it (F.S.); giuseppe.ricci@burlo.trieste.it (G.R.); 2 Department of Biomedical Sciences, University of Padova, Padova 35121, Italy; giorgio.arrigoni@unipd.it (G.A.); cinzia.franchin@unipd.it (C.F.); 3 Proteomics Center, Padova University, Padova 35129, Italy; 4 Department of Medical, Surgery and Health Sciences, University of Trieste, Trieste 34149, Italy

**Keywords:** leiomyoma, 2-DE, myometrium, metabolic process, proteomic

## Abstract

Uterine leiomyoma is the most common benign smooth muscle cell tumor of the uterus. Proteomics is a powerful tool for the analysis of complex mixtures of proteins. In our study, we focused on proteins that were upregulated in the leiomyoma compared to the myometrium. Paired samples of eight leiomyomas and adjacent myometrium were obtained and submitted to two-dimensional gel electrophoresis (2-DE) and mass spectrometry for protein identification and to Western blotting for 2-DE data validation. The comparison between the patterns revealed 24 significantly upregulated (*p* < 0.05) protein spots, 12 of which were found to be associated with the metabolic processes of the leiomyoma and not with the normal myometrium. The overexpression of seven proteins involved in the metabolic processes of the leiomyoma was further validated by Western blotting and 2D Western blotting. Four of these proteins have never been associated with the leiomyoma before. The 2-DE approach coupled with mass spectrometry, which is among the methods of choice for comparative proteomic studies, identified a number of proteins overexpressed in the leiomyoma and involved in several biological processes, including metabolic processes. A better understanding of the mechanism underlying the overexpression of these proteins may be important for therapeutic purposes.

## 1. Introduction

Uterine leiomyoma is the most common benign smooth muscle cell tumor of the uterus [1]. This benign tumor is responsible for several problems related to the female reproductive tract, resulting in excessive uterine bleeding, pressure on adjacent organs, infertility and recurrent pregnancy loss [2]. The causes of leiomyoma growth are still unknown, as are those relevant for the malignant transformation in leiomyosarcomas [3]. Metabolic differences might be responsible for the increase of the estrogenic effect, inducing a higher risk for clinically-significant fibroids in African-American women [4]. Fibroids are associated with metabolic syndrome and, thus, with abnormal glucose metabolism [5]. Metformin inhibits the growth of the leiomyoma, and this effect is mediated by the activation of the AMPK and by the subsequent inhibition of the mammalian target of rapamycin (mTOR) pathway [6].

Transforming growth factor beta (TGF-β), basic fibroblast growth factor (bFGF) and platelet-derived growth factor (PDGF) play an important role in cell proliferation and in the production of the extracellular matrix (ECM) in leiomyoma growth [7]. Microarray analysis of 22,283 genes in paired samples of leiomyoma and adjacent normal myometrium identified several gene expression dysregulations, including retinoid synthesis, insulin-like growth factor (IGF) metabolism, TGF-β signaling and extracellular matrix formation [8]. Turunen *et al.* described how uterine leiomyoma-linked mutations in MED12 lead to a highly specific decrease in its association with cyclin C-CDK8/CDK19 and to loss of mediator-associated cyclin-dependent kinase (CDK) activity. This finding indicates that the MED12/cyclin C interface is a potential therapeutic target in CDK8-driven cancers [9]. Cellular retinoic acid-binding protein 2 (CRABP2) and epidermal fatty acid-binding protein (FABP5) regulate the partition of retinoic acid (RA) in its two receptors: RAR and PPARβ/δ [10]. FABP5/PPARβ/δ are known to be oncogenes, and the inhibition of their transcriptional function can be used as a strategy in the treatment of breast cancer [11]. Glycolysis is increased in breast adenocarcinoma, and GOT1 is a key glycolytic enzyme [12]. Studies on the activity of GOT1 in breast cancer have identified it as a potential molecular target for the development of anti-neoplastic agents [12].

A previously-published study on leiomyoma interstitial fluid (IF) identified a number of dysregulated proteins with possible involvement in cell proliferation and ECM accumulation and, thus, in leiomyoma growth [13].

Proteomics is a powerful tool for the analysis of complex mixtures of proteins. Lemeer *et al.* performed quantitative proteome and kinome profiling of leiomyoma *vs.* myometrium using GeLC-MS/MS, identifying several dysregulated kinases [14]. The present study aimed at identifying upregulated proteins related to molecular mechanisms involved in leiomyoma development. We identified several upregulated proteins involved in metabolic and other biological processes. The identified proteins could be involved in processes that lead to tumor growth. Further studies are necessary to understand metabolic dysregulation in leiomyomas.

## 2. Result

### 2.1. Proteomic Studies

**Figure 1:**
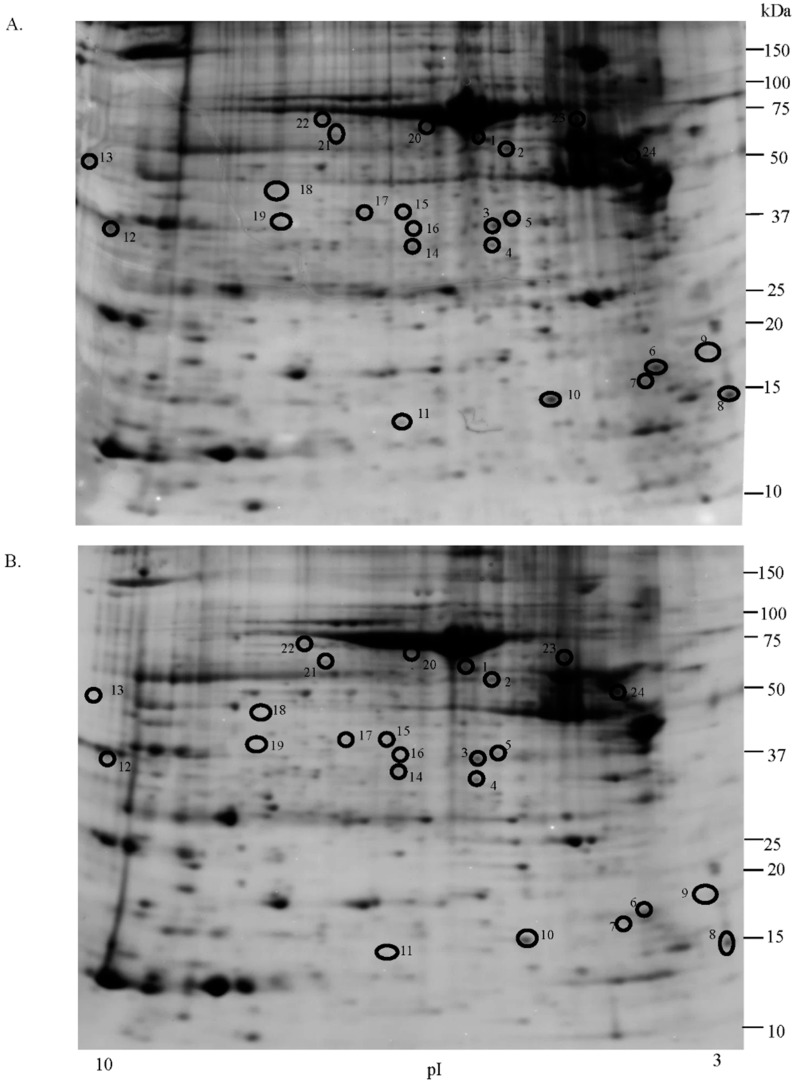
Two-dimensional electrophoresis map of uterine leiomyoma (**A**) and normal myometrium (**B**) proteome. Black circles indicate up-regulated protein spots. Immobilized pH gradient 3–10 NL strips were used for the first dimension, and 12.5% polyacrylamide gel was used for the second dimension.

Comparative proteomic analysis was performed between uterine leiomyoma and myometrium tissues in order to generate 2-DE reference maps and to identify upregulated proteins. An average of 2000 spots was detected on gels for both types of proteomes. Analyses indicate that 24 protein spots were significantly upregulated in leiomyoma samples when compared to the myometrium (*p* < 0.05; in terms of expression, all 24 with a fold change ≥1.5-fold) (Figure 1; Figures S1 and S2). We also identified four downregulated proteins (transgelin, lamin A/C, α-1-actinin and carbonic anhydrase 1), but the differences were not significant and were thus not investigated further.

The 24 protein spots were identified using MALDI-TOF/TOF and LTQ-Orbitrap XL, searching the MS/MS data against the human section of the UniProt database (Version 20140709, 88,993 sequences) (Table 1; Tables S1 and S2). Considering that 24 tests were carried out, one for each protein, we also report the *p*-values adjusted according to the Bonferroni correction for multiple comparisons. We are aware that applying the Bonferroni correction, none of the adjusted *p*-values remains statistically significant.

Among the proteins identified, four had never previously been associated with leiomyoma: isoform 2 of guanine nucleotide-binding protein G(I)/G(S)/G(T) subunit β-1, isoform 3 of polymerase I and transcript release factor, isoform 5 of prelamin-A/C and FHL1. Proteins GOT1, FABP5, CRABP2, isoform 2 of guanine nucleotide-binding protein G(I)/G(S)/G(T) subunit β, isoform 3 of polymerase I and transcript release factor and isoform 5 of prelamin-A/C and FHL1 were further validated to confirm 2-DE data. The remaining 20 proteins have already been shown to be associated with leiomyoma using GeLC-MS/MS [14].

### 2.2. Immunochemical Study of Proteins Expression

**Figure 2:**
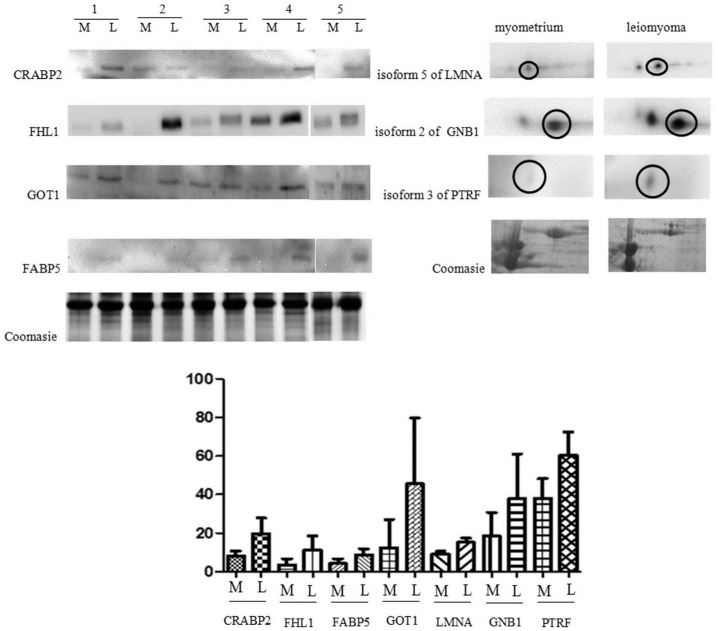
Western blot analysis of CRABP2, FHL1, FABP5, GOT1, isoform 5 of LMNA, isoform 2 of GNB1 and isoform 3 of PTRF in paired myometrium (M) and leiomyoma (L). The isoform corresponding to the one identified by 2-DE is circled. The intensity of immunostained bands was normalized against the total protein intensities measured from the same blot stained with Coomassie brilliant blue. The bar graph shows the relative expression (band density) of proteins in the myometrium and the leiomyoma. The results are shown as a histogram (mean) with whiskers representing the standard deviation. All differences were found to be significant (Wilcoxon signed-rank test for matched samples, *p* < 0.05).

In this study, western blot (WB) analysis was used to validate the expression of CRABP2, FHL1, FABP5, GOT1 and 2D Western blotting for isoform 2 of guanine nucleotide-binding protein G(I)/G(S)/G(T) subunit β, isoform 3 of polymerase I and transcript release factor and isoform 5 of prelamin-A/C, in five leiomyomas compared to matched normal myometrial tissue. 2D Western blotting was used to validate the three isoforms, because a normal WB would not have allowed distinguishing between the different isoforms (all with very similar molecular weight), since the antibodies were not raised against one of the specific isoforms of the proteins. Therefore, the only way to distinguish between them was to separate the different isoforms by 2-DE prior to the immunochemical reaction [15]. Western blotting and 2D Western blotting protein expressions were significantly higher in the leiomyoma compared to the myometrium, confirming the results obtained from the 2-DE analysis (Figure 2).

The specific isoforms identified by 2-DE showed the same trend of change in the leiomyoma when analyzed by 2D Western blotting. Among the train of spots recognized as LMNA, one corresponding to spot 22 (isoform 5 of LMNA) appeared more intense in leiomyoma then in myometrium. Similarly, among the spots recognized as GNB1, one corresponding to spot 5 (isoform 2 of GNB1) was increased in leiomyoma. Finally, isoform 3 of PTRF was validated by 2D Western blotting confirming the 2-DE data (Figure 2).

To normalize the results of our WB analysis, we determined the total protein content of each sample by Coomassie staining. The reason for this was that, according to our results, the proteins that are usually selected as encoded by housekeeping genes (*i.e.*, β-actin and tubulin) are upregulated in leiomyoma and, thus, not adequate to be used as controls for normalization. Because we could not establish which proteins should be considered as housekeeping in our samples, we decided to apply a total protein content normalization method, as described by Lv *et al.* and Lemeer *et al.* [3,14].

### 2.3. Protein–Protein Interaction and Co-Expression Study

**Figure 3:**
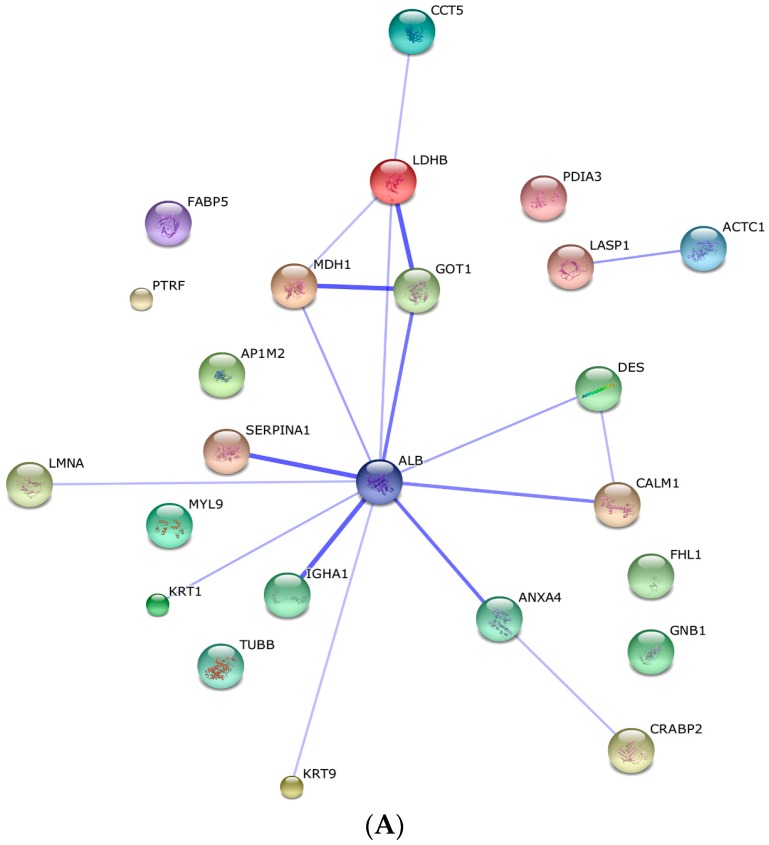

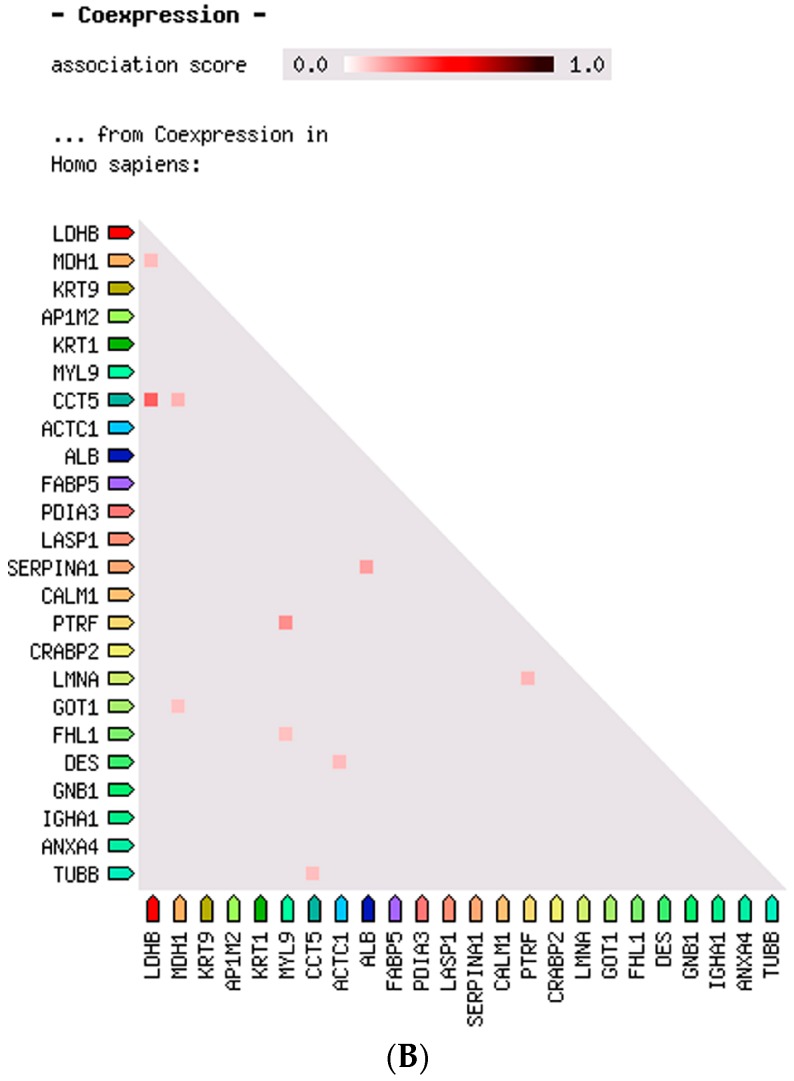
Prediction by STRING database: (**A**) protein interactions based on confidence prediction and (**B**) co-expression of upregulated proteins in the leiomyoma.

The STRING software elaborated a protein–protein interaction network based on confidence prediction. Further, leiomyoma protein–protein interaction was analyzed for co-expression mapping. There appears to be an interesting strong association among the genes of three metabolic enzymes: between LDHB and GOT1 (combined score: 0.964) and between GOT1 and MDH1 (combined score: 0.982). We further analyzed the co-expression mapping (Figure 3), and the most significant co-expression was found between CCT5 and LDHB. In addition, PTRF co-expresses with KRT1, CCT5 co-expresses with MHI1; GOT1 co-expresses with MHI1; and MHI1 co-expresses with LDHB; indicating not only an interaction between LDHB, GOT1 and MDH1, but also a possible association between them and the leiomyoma.

### 2.4. Functional Analysis of the Leiomyoma Proteome

**Figure 4:**
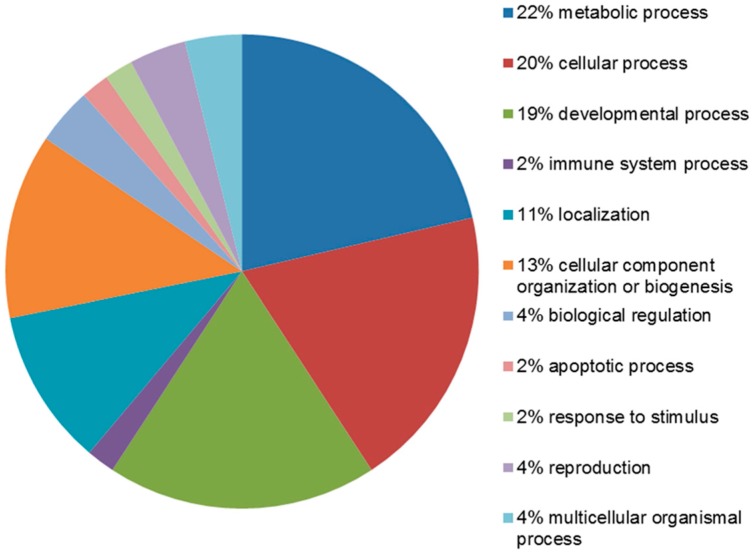
PANTHER classification of proteins upregulated in leiomyoma according to their biological process.

The identified proteins were divided into groups, based on the PANTHER classification system and using the STRAP 5.1 software, according to their biological processes and cellular composition. In terms of biological processes, the proteins were grouped into four main categories: metabolic process, cellular process, developmental process and cellular component organization or biogenesis (Figure 4).

**Figure 5:**
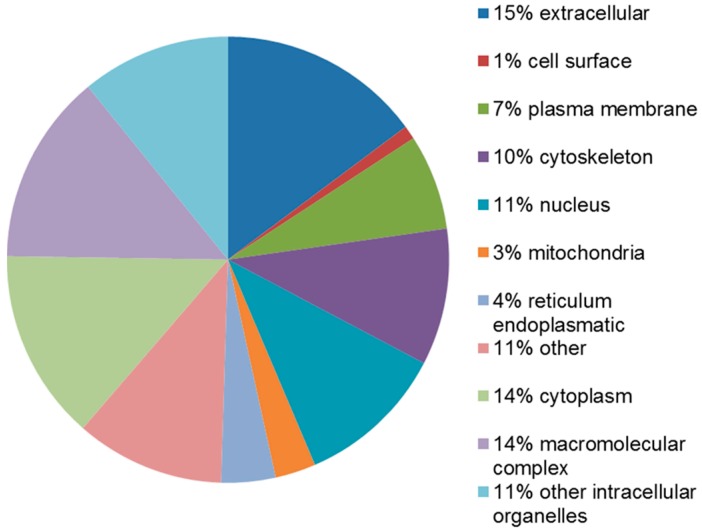
STRAP classification of proteins upregulated in the leiomyoma according to their subcellular localization.

Twelve proteins (PDIA3, CCT5, LDHB, isoform 2 of guanine nucleotide-binding protein G(I)/G(S)/G(T) subunit β-1, MDH1, GOT1, ANXA4, CRABP2, isoform 3 of polymerase I and transcript release factor, FABP5, FHL1 and SERPINA1) were found to be associated with the metabolic process, according to the PANTHER prediction. The STRAP software instead locates these proteins mainly in the extracellular region, cytoplasm, macromolecular complex, cytoskeleton and in other intracellular organelles (Figure 5).

## 3. Discussion

Several cytogenetic studies, microarray investigations and proteomic studies of the human leiomyoma *vs.* the unaffected uterine myometrium have been conducted to try to understand the pathogenesis of leiomyoma [11,12].

We employed a classical approach based on 2-DE, coupled with mass spectrometry, in order to analyze the leiomyoma proteome and to identify proteins that are significantly upregulated in the leiomyoma compared to the myometrium. With this approach, we identified 24 upregulated proteins, 12 of which were involved in the metabolic process. CRABP2, FHL1, FABP5, GOT1, isoform 5 of LMNA, isoform 2 of GNB1 and isoform 3 of PTRF were further validated by Western blot and 2D Western blotting to confirm the 2-DE result. The 2-DE approach made it possible to detect several proteins with increased expression, compared to normal myometrium, never previously reported in the literature of the leiomyoma.

In leiomyoma, glycolytic enzymes are upregulated in order to produce the ATP and anabolic precursors required for cell proliferation. In particular, the expression of LDHB regulates the glycolytic flux by converting pyruvate to lactate, and indeed, increased LDHB expression has been observed in breast cancer [16].

GOT1 is involved in amino acid metabolism and in the biosynthesis of l-glutamate from l-aspartate or l-cysteine [17]. Increased serum fatty acid levels cause insulin resistance and diabetes [17]. It is quite clear that GOT1 is involved in the regulation of fatty acid levels [16], and this would support the hypothesis of a possible involvement of GOT1 in the association between metabolic syndrome and leiomyoma growth. This enzyme is required to shuttle electrons from glycolytic cytoplasmic NADH to mitochondrial NADH. The malate-aspartate shuttle is active in several tumors and accounts for 20% of the total respiratory rate [12]. Interestingly, our STRING interaction network and gene co-expression data are confirmed in the literature. Oxamate inhibits GOT1, disrupting the growth of breast adenocarcinomas [12]. A better understanding of the regulation of glucose metabolism in leiomyoma may help identify new metabolic targets for tumor treatment.

Increased levels of estrogen induce overexpression of CRABP2, leading to increased expression of retinoic acid (RA). This causes the activation of HMG (high-mobility group) genes, resulting in the under-expression of the enzymes involved in retinoid synthesis and cell differentiation. In addition, RA induces overexpression of MMP11, which degrades IGF-binding proteins and may lead to cell proliferation, decreased apoptosis and accelerated ECM accumulation, thereby promoting the growth of uterine leiomyomas [8,18]. FABP5 transports RA to the nucleus and delivers it to PPARβ/δ, activating non-genomic pathways, leading to proliferative and antiapoptotic effects on cancer cells [19]. A similar mechanism may occur in leiomyomas.

PDIA3 can modulate the levels and activity of 17-β estradiol (E2) in breast cancer cells and may have a proliferative effect, promoting tumor growth [20].

CCT5 is part of the CCT/TRiC complex, which contains eight different subunits, including subunit ε [20]. CCT/TRiC mediates the folding of tubulins and actins [21], plays a key role in cell cycle progression and could be implicated in tumor development [22].

In our study, we observed the upregulation of FHL1, a protein that mediates signaling, regulates the cytoskeleton-associated protein–protein interaction of transcription factors and has never previously been described as associated with leiomyomas. Overexpression of FHL1 in Sol8 myoblast inhibits cell adhesion and promotes cell migration and proliferation [23]. A study on the methylation of DNA in leiomyoma revealed hypomethylation of the FHL1 gene in leiomyoma [24], meaning that a transcriptional profiling of the leiomyoma and of the unaffected myometrium revealed an upregulation of FHL1 m-RNA in the leiomyoma [25], which is in agreement with our data on protein expression.

A number of studies have shown that ANXA4 takes part in several biological functions in a Ca^2+^-dependent manner, including apoptosis and growth control [26], and that it is overexpressed in ovarian [27] and breast cancers [28]. The results of these studies are in agreement with our findings.

Lemeer *et al.* [14] conducted a detailed study on the proteomic profile of leiomyoma and myometrium samples from eight patients using label-free GeLC-MS/MS, which resulted in the identification and intensity-based label-free quantification of more than 7000 proteins.

We compared our data to their data. The comparison confirmed that the expression of TUBB, KRT1, LASP1, ACTC1, MDH1, LDHB, CCT5, PDIA3, KRT9, AP1M2, GOT1, FABP5 and CRABP2 was upregulated, while the expression of LMNA, TAGLN, ACTN1 and CA1 was downregulated. In our previous study, DES and SERPINA1 were validated by immunohistochemistry as upregulated proteins, which is in line with our present data [13]. Leiomyoma is a hormonal-dependent tumor, and the expression of proteins is strongly related to hormonal levels [29].

As mentioned above, CCT/TRiC mediates the folding of TUBB, which interacts with ERα through HPIP (hematopoietic PBX-interaction protein). The destabilization of the microtubules induces activation of ERα signaling, which indicates the implication of the HPIP-microtubules complex [30], leading to leiomyoma growth. On the other hand, 2-methoxyestradiol (2ME) microtubule-targeting agent represents a potential new therapeutic target for leiomyoma [31].

DES overexpression in the cell and in the IF can induce an alteration of the mechanotransductional signal affecting leiomyoma growth [12]. KRT1 may regulate the activity of kinase PKC [32], which is involved in leiomyoma growth control [33].

CALM1 mediates the control of a large number of enzymes, such as protein kinases, phosphatases and other proteins, via Ca^2+^ [34,35]. Steroidal hormones, growth factors and cytokines stimulate the Ca^2+^/CALM1-dependent pathway, leading to cell proliferation [36].

ACTC1 belongs to the actin family, whose members are highly conserved proteins involved in various types of cell motility and development [37]. An increase in viscoelastic forces is associated with alteration of cell structure and actin organization, which lead to increased expression of several types of actins, including ACTC1, inducing leiomyoma growth [13,38].

AP1M2 mediates the recruitment of clathrin to membranes and the recognition of sorting signals within the cytosolic tails of transmembrane cargo molecules [39]. Several studies indicate that under hypoxia, AP1M2 promotes angiogenesis in different cancer cell lines [40].

LASP1 contains a C-terminal src homology SH3 domain, involved in the interaction with zyxin, which functions as a messenger in the signal transduction pathway and may modulate the cytoskeletal organization of actin bundles [41]. Overexpression of LASP1 in breast cancer may regulate gene transcription by recruiting zyxin to focal adhesions and induce tumor cell growth and migration [41]. These results are in agreement with what we observed in the leiomyoma and in the normal myometrium.

Unfortunately, we did not detect PCNA and collagens. The reason for not detecting PCNA could be that its concentration was too low for it to be visible on 2-DE; while the reason for not detecting collagens could lay either in the fact that these are secreted proteins or that their molecular weight is too high for them to transfer from the IPG strip to SDS-PAGE [42].

Three protein isoforms were identified with 2-DE in our study: isoform 2 of guanine nucleotide-binding protein G(I)/G(S)/G(T) subunit β-1, isoform 3 of polymerase I and transcript release factor and isoform 5 of prelamin-A/C. The presence of isoforms is probably due to alternative splicing, and these may play different roles inside the cells with possible implications on the mechanisms involved in cell proliferation. Further studies will be needed to investigate the role of these upregulated proteins in leiomyoma and their association with tumor growth, decreased apoptosis and increased ECM production. Since 12 of the upregulated proteins we detected are involved in metabolic processes, we believe that further research into metabolites would help to better understand the metabolic processes involved in the physiopathology of the tumor.

## 4. Experimental Section

In order to identify upregulated proteins in the leiomyoma proteome, eight pairs of normal myometrium and leiomyoma tissue samples from individual patients were subjected to 2-DE analysis combined with protein identification by MALDI-TOF/TOF and the LTQ-Orbitrap XL mass spectrometer.

Tissue samples were obtained from eight premenopausal patients who underwent hysterectomy for symptomatic uterine leiomyomas. The procedures complied with the Declaration of Helsinki and were approved by the review board of the Institute for Maternal and Child Health-IRCCS “Burlo Garofolo” of Trieste, Italy. All subjects signed written informed consent. All samples were taken during the proliferative phase. Hormonal levels can modify the expression of proteins, as described in a previous study published by our research group [29]. The median age of patients was 47 with a minimum of 41 years and a maximum of 52 years.

### 4.1. Tissue Samples

Two samples were collected from each patient: one from the central area of the leiomyoma and one from the unaffected myometrium. All leiomyomas were confirmed histologically as benign ordinary leiomyomas. Samples were stored at −80 °C until proteomic analysis was performed. Pictures of samples from the leiomyoma and myometrium of two different patients are shown as the Supplementary Material.

### 4.2. Two-Dimensional Gel Electrophoresis

Sampling for 2-DE analysis was performed as previously described [43]. Clean leiomyoma and myometrium (300 mg each) were homogenized in 1.2 mL of dissolution thiourea/urea/CHAPS (TUC) buffer (7 M urea, 2 M thiourea, 4% CHAPS, 40 mM Tris, 65 mM DTT and 0.24% Bio-Lyte (3-10)) (Sigma-Aldrich, Saint Louis, MO, USA) with a protease inhibitor mix (2 mM phenylmethylsulfonyl fluoride (PMSF), 1 mM benzamidine, 1 mM EDTA, 1 mM NaF) (Sigma-Aldrich) and a trace of bromophenol blue. The solutions were vortexed at maximum speed several times and kept at room temperature for 1 h and centrifuged at 14,000× *g* at 4 °C for 30 min. The protein content of the supernatant was determined using the Bradford assay. One thousand micrograms of proteins from each sample were used for 2-DE analysis.

Seventeen-centimeter pH 3–10 NL (IPG) Readystrips (Bio-Rad, Hercules, CA, USA) were rehydrated at 50 V for 12 h at 20 °C. Isoelectric focusing (IEF) was performed in a Protein IEF Cell (Bio-Rad) set to 160,000 Vh. After IEF, the IPG strips were equilibrated by serial incubation (20 min) in equilibration buffer (6 M urea, 2% SDS, 50 mM Tris-HCl pH 8.8, 30% glycerol and 1% DTT) and in equilibration buffer containing 4% iodoacetamide instead of DTT. Equilibrated IPG strips were transferred onto a 12.5% polyacrylamide gel for the second dimension. The second dimension was run on Protean II-XL Cell (200 V constant voltage) until the bromophenol blue reached the bottom of the gel. After overnight protein fixation in 40% methanol and 10% acetic acid, gels were washed twice for 20 min in distilled water. The gels were stained for 48 h with colloidal Coomassie blue, and the excess of dye was removed with distilled water. On average, three experimental replicates were performed per sample. Molecular masses were determined by precision protein standard markers (Bio-Rad), covering the range of 10–250 kDa. 2-DE gels were scanned with a Molecular Imager PharosFX System (Bio-Rad). The reproducibility of protein profiles was good with average matching efficiency of about 75%, and the quantitative analysis of the spots was carried out using the Proteomweaver 4 program (Bio-Rad).

### 4.3. Quantification of Spot Levels

Spot quantification was performed as previously described [44]. 2-DE image analysis was performed using Proteomweaver 4 software (Bio-Rad). The analysis process was carried out by matching all gels from eight myometria and eight leiomyomas. The Proteomweaver 4 algorithm matched all of the gels to find quantitative differences. Differences were considered significant when the ratio of the mean percentage relative volume (%*V*) (%*V* = *V*(single spot)/*V*(total spot)) was ±1.5-fold and satisfied the non-parametric Wilcoxon test (*p* < 0.05). Only the spots with the same changing trend in all three gels were considered for further analysis. The fold change was calculated as the ratio between the mean percentage relative volume of the uterine leiomyoma and the normal myometrium.

### 4.4. Trypsin Digestion

Spots from 2D-gel were washed four times with 50 mM NH_4_HCO_3_ and acetonitrile (ACN) (Sigma-Aldrich) alternatively and dried under vacuum in a SpeedVac system. Three microliters of 12.5 ng/μL sequencing-grade modified trypsin (Promega, Madison, WI, USA) in 50 mM NH_4_HCO_3_ were added to each gel spot, and samples were digested overnight at 37 °C. Peptides were extracted with 3 changes of 50% ACN/0.1% formic acid (FA, Fluka, Sigma-Aldrich, Milano, Italy), dried under vacuum and stored at −20 °C until MS analysis was performed.

### 4.5. MS Analysis

Samples were dissolved in 10 μL of 0.1% trifluoroacetic acid (TFA, Riedel-de Haën, Hanover, Germany). One microliter of each sample was mixed with one μL of matrix solution (α-cyano-4-hydroxycinnamic acid (Fluka) at a concentration of 5 mg/mL in 70% ACN/0.1% TFA). Zero-point-eight of the resulting solution were spotted onto a stainless steel MALDI target plate for MS analysis on the MALDI-TOF/TOF 4800 analyzer (AB Sciex, Framingham, MA, USA). The analysis was performed in a data-dependent mode: a full MS scan was acquired from each sample, followed by MS/MS spectra of the ten most intense signals.

Samples that could not be identified by MALDI-TOF/TOF analysis were further analyzed by LC-MS/MS on an LTQ-Orbitrap XL mass spectrometer (ThermoFisher Scientific, Rockford, IL, USA), coupled with a nano-HPLC Ultimate 3000 (Dionex-ThermoFisher Scientific). Samples were loaded onto a homemade pico-frit column packed with C18 material (ReproSil, 300 Å, 3 μm; Dr. Maisch HPLC GmbH, Ammerbuch, Germany) and separated using a 20-min linear gradient of ACN/0.1% formic acid (from 3%–40% ACN), at a flow rate of 250 nL/min. The capillary voltage was set at 1.3–1.5 kV and the source temperature at 200 °C. The analysis was performed in a data-dependent mode, and the full scan at 60,000 resolution on the Orbitrap was followed by MS/MS fragmentation scans on the ten most intense ions acquired with collision-induced dissociation (CID) fragmentation in the linear trap.

MS and MS/MS spectra obtained from MALDI-TOF/TOF analysis were converted into MGF (Mascot Generic Format) files to be processed with Proteome Discoverer 1.4 (ThermoFisher Scientific), while raw data files from the LTQ-Orbitrap XL mass spectrometer were directly analyzed with the software. Proteome Discoverer was interfaced to a Mascot search engine, Version 2.2.4 (Matrix Science, London, UK).

The database used for protein identification was UniProt Human (Version 20140709, 88,993 sequences), while enzyme specificity was set to trypsin with 1 missed cleavage. The mass tolerance window was set to 10 ppm for parent mass and to 0.6 Da for fragment ions, for files generated by the LTQ-Orbitrap XL, and at 50 ppm (parent) and 0.3 Da (fragment ions) for MALDI-TOF/TOF data. Carbamidomethylation of cysteine residues was set as fixed modification and methionine oxidation as variable modification.

Proteome Discoverer calculates a false discovery rate (FDR) based on the parallel search against a randomized database. Proteins were considered as positive hits if at least two independent peptides were identified with medium (95%) or high (99%) confidence.

### 4.6. Protein–Protein Interaction, Co-Expression and Functional Analysis

Upregulated proteins identified by MS in the leiomyoma were uploaded onto STRING 9.0 (Search Tool for the Retrieval of Interacting Genes) database search. The different expression proteins thus identified were analyzed by PANTHER 9.0 (Protein Analysis through Evolutionary Relationships; http://www.pantherdb.org) and STRAP 1.5 (Software Tool for Rapid Annotation of Proteins: Cardiovascular Proteomics Center, Boston University School of Medicine, Boston, MA, USA). Proteins were then classified according to their involvement in biological processes and their subcellular localization. As most of the proteins take part in multiple processes, only the most relevant are reported.

### 4.7. Western Blot Analysis

Western blot analysis was performed as previously described [45]. Protein extracts (30 μg) used for 2-DE were separated by 10% SDS-PAGE and then transferred to a nitrocellulose membrane in a blotting chamber. For 2D Western blotting, 200 μg of protein extract were treated as for 2-DE (described above) and then subjected to the blotting procedure. The residual binding sites on the membrane were blocked by treatment with defatted dry milk proteins and incubated overnight at 4 °C with 1:500 diluted primary rabbit polyclonal antibody against aspartate aminotransferase, cytoplasmic, 1:500 diluted primary rabbit polyclonal antibody against cellular retinoic acid-binding protein 2, 1:200 diluted primary rabbit polyclonal antibody against four and a half LIM domains protein 1, 1:200 diluted primary rabbit polyclonal antibody against epidermal fatty acid binding protein, 1:1000 diluted primary rabbit polyclonal antibody against guanine nucleotide-binding protein G(I)/G(S)/G(T) subunit β, 1:200 diluted primary rabbit polyclonal antibody against prelamin-A/C, 1:500 diluted primary rabbit polyclonal antibody against polymerase I and transcript release factor (Sigma-Aldrich). After washing, membranes were incubated with a HRP-conjugated anti-rabbit IgG (Sigma-Aldrich) in a dilution of 1:3000.

Finally, the protein expression was visualized by chemiluminescence (Super Signal West Pico Chemiluminescent, Thermo Fisher Scientific) according to the manufacturer’s instructions. The intensity of the signals was quantified by a Versa-Doc Imaging System (Bio-Rad). The intensities of the immunostained bands were normalized with the total protein intensities measured by Coomassie brilliant blue G-250 from the same blot.

### 4.8. Statistical Analyses

Statistical analyses were carried out with the non-parametric Wilcoxon signed-rank test for paired samples for 2-DE, Western blot and 2D Western blotting data. A *p*-value <0.05 was considered as statistically significant. Analyses were conducted with Stata/IC 12.2 for Windows (Stata Corp LP, College Station, TX, USA).

## 5. Conclusions

The 2-DE approach coupled with MS is a powerful tool for comparative proteome studies and for the identification of protein isoforms. In the present study, we identified several upregulated proteins involved in metabolic and other biological processes. The identified proteins could be involved in processes that lead to tumor growth. Further studies are necessary to understand metabolic dysregulation in leiomyomas. A better understanding of the mechanisms underlying the overexpression of these proteins may be important for therapeutic purposes.

## Figures and Tables

**Table 1:** Protein expression levels measured in the leiomyoma and in the myometrium proteome as identified by MALDI-TOF/TOF or LTQ-Orbitrap XL mass spectrometer and classified by their corresponding biological process and subcellular localization. Only the most relevant biological processes are reported.

Accession Number	Spot No.	Protein Description	Gene Symbol	Protein Score *	Fold Change **	SEM ***	Subcellular Localization	*p*-Value	Adjusted *p*-Value ****
Metabolic process									
Lipid metabolic process									
Q01469	11	Fatty acid binding protein, epidermal	FABP5	348	3.5	1.220	Cytoplasm	0.011	0.264
P29373	10	Cellular retinoic acid-binding protein 2	CRABP5	145	2.25	0.397	Cytoplasm	0.011	0.264
P09525	4	Annexin A4	ANXA4	2767	1.5	0.202	Extracellular	0.029	0.696
Carbohydrate and TCA metabolic process									
P40925	19	Malate dehydrogenase cytoplasmic	MDH1	45	2.4	0.691	Cytoskeleton	0.022	0.528
P07195	3	l-lactate dehydrogenase B chain	LDHB	1526	1.5	0.241	Cytoplasm	0.014	0.336
Amino acid metabolic process									
P17174	18	Aspartate aminotransferase cytoplasmic	GOT1	225	4.3	0.444	Cytoplasm	0.018	0.432
Nucleobase-containing compound metabolic process									
Q13642	12	Four and a half LIM domains protein 1	FHL1	224	5	2.338	Cytoplasm	0.022	0.528
P62873	5	isoform 2 of Guanine nucleotide-binding protein G(I)/G(S)/G(T) subunit β	GNB1	114	1.76	0.266	Other cell body	0.018	0.432
Q6NZI2	9	isoform 3 of polymerase I and transcript release factor	PTRF	229	1.75	0.793	Plasma membrane	0.019	0.456
Protein metabolic process									
P48643	17	T-complex protein 1 subunit epsilon	CCT5	60	2.5	1.184	Other cell body	0.035	0.840
P30101	1	Protein disulfide isomerase A3	PDIA3	161	1.6	0.359	Cell surface	0.019	0.456
P09525	24	α-1-antitrypsin	SERPINA1	335	1.5	0.832	Endoplasmatic reticulum	0.014	0.336
Cellular process									
Cell communication, cycle and movement									
P68032	23	Actin, α cardiac muscle 1	ACTC1	248	5.1	1.527	Cytoskeleton	0.018	0.432
P04264	13	Keratin, type II cytoskeletal 1	KRT1	107	3.1	1.821	Extracellular	0.022	0.528
P02545	8	Calmodulin 1	CALM1	313	1.9	0.258	Cytoskeleton	0.011	0.264
P07437	2	Tubulin β chain	TUBB	317	1.9	0.455	Cytoskeleton	0.011	0.264
Developmental process									
Anatomical structure and morphogenesis									
P02545	22	isoform 5 of prelamin-A/C	LMNA	128	2.4	1.043	Cytoplasm	0.028	0.672
P35527	14	Keratin, type I cytoskeletal 9	KRT9	214	1.9	0.288	Extracellular	0.035	0.840
Cellular component organization or biogenesis									
Cellular component organization									
P24844	6	Myosin regulatory light polypeptide 9	MYL9	100	2	0.482	Cytoplasm	0.017	0.408
P17661	7	Desmin	DES	190	1.7	0.626	Cytoplasm	0.035	0.840
Transport									
P02768	21	Serum albumin	ALB	117	2.5	0.803	Extracellular	0.018	0.432
Q9Y6Q5	16	AP-1 complex subunit mu-2	AP1M2	54	2.2	0.720	Macromolecular complex	0.045	1.080
Immune system process									
P01876	20	Immunoglobulin heavy constant α 1	IGAH1	68	1.9	0.412	Extracellular	0.028	0.672
Biological regulation									
Q14847	15	LIM and SH3 domain protein 1 fragment	LASP1	66	1.7	0.444	Cytoskeleton	0.031	0.744

* The protein score is the sum of the Mascot ion scores of all of the peptides that were identified for a given protein; ** the fold change is the ratio of the mean percentage relative volume (%*V*) (%*V* = *V*(single spot)/*V*(total spot)) of the uterine leiomyoma and the normal myometrium; *** SEM: standard error of the mean; **** *p*-value adjusted according to the Bonferroni correction: the *p*-value was multiplied by the number of tests simultaneously carried out (*n* = 24).

## Supplementary Materials

Supplementary materials can be found at http://www.mdpi.com/1422-0067/17/4/540/s1.
