# Supplementary material for: A Proteomic Approach for the Identification of Up-Regulated Proteins Involved in the Metabolic Process of the Leiomyoma
Source: Int J Mol Sci. 2016 Apr 9;17(4):540. doi: 10.3390/ijms17040540 (PMC4848996; doi:10.3390/ijms17040540)

Figure S1: Visualization of spots for up-regulated proteins.

Myometrium Leiomyoma

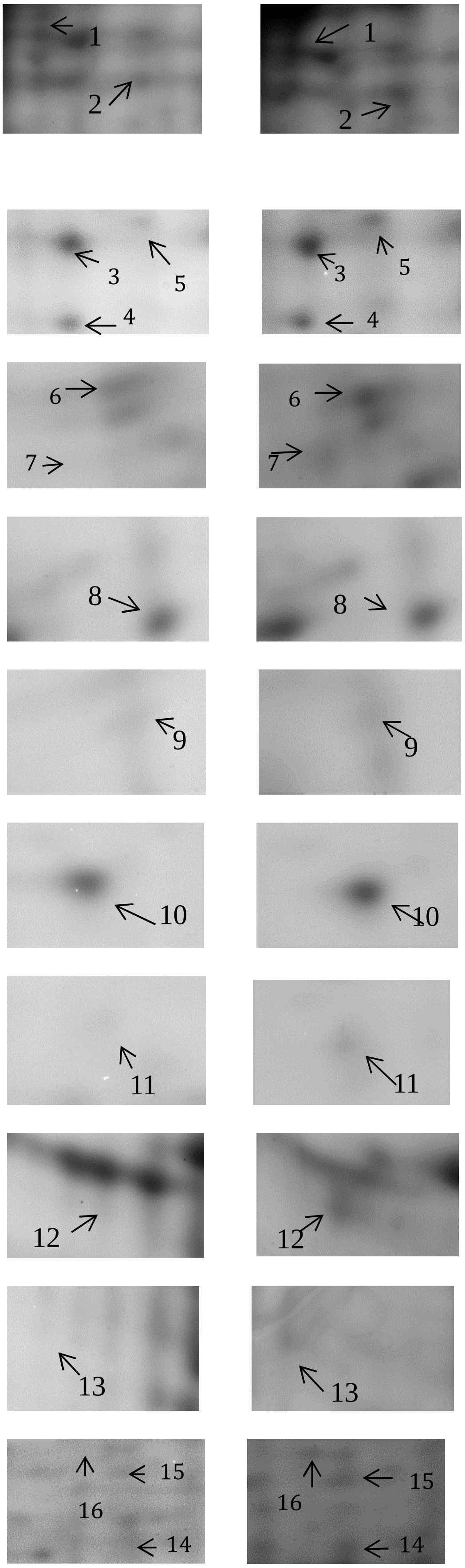

1. Protein disulfide isomerase A3
2. Tubulin β chain

3. L-lactate dehydrogenase B chain
4. Annexin A4
5. isoform 2 of Guanine nucleotide-binding protein G(I)/G(S)/G(T) subunit β

6. Myosin regulatory light polypeptide 9
7. Desmin

8. Calmodulin 1

9. Isoform3 of Polymerase I and transcript

10. Cellular retinoic acid-binding protein 2

11. Fatty acid binding protein, epidermal

12. Four and a half LIM domains protein 1

13. Keratin, type II cytoskeletal 1

14. Keratin, type I cytoskeletal 9
15. LIM and SH3 domain protein 1 fragment
16. AP-1 complex subunit mu-2

17. T-complex protein 1 subunit epsilon

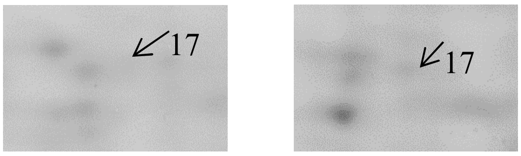

18. Aspartate aminotransferas cytoplasmatic

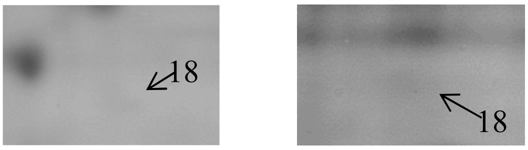

19. Malate dehydrogenase cytoplasmic

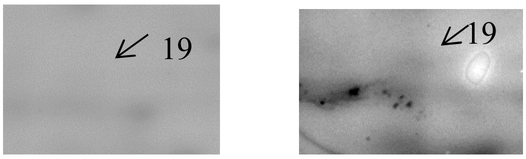

20. Immunoglobulin heavy constant α 1

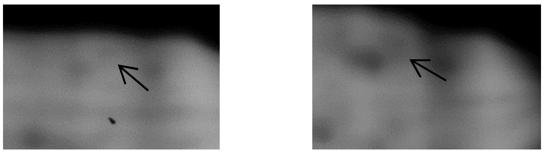

21. Serum albumin
22. isoform 5 of Prelamin-A/C

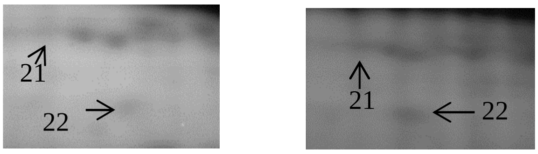

23. Actin, α cardiac muscle 1

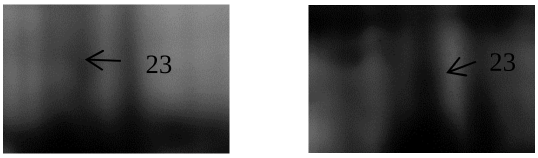

24. α-1-antitrypsin

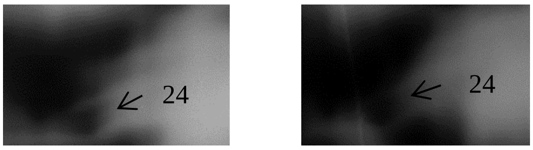

Supplement: Supplementary file 1 [file ijms-17-00540-s001.zip › ijms-109333-supplementary-revise 1/Figure S1.pdf]
