# Supplementary material for: A Proteomic Approach for the Identification of Up-Regulated Proteins Involved in the Metabolic Process of the Leiomyoma
Source: Int J Mol Sci. 2016 Apr 9;17(4):540. doi: 10.3390/ijms17040540 (PMC4848996; doi:10.3390/ijms17040540)
Supplement: Supplementary file 1 [file ijms-17-00540-s001.zip › ijms-109333-supplementary-revise 1/Figure S3.pdf]

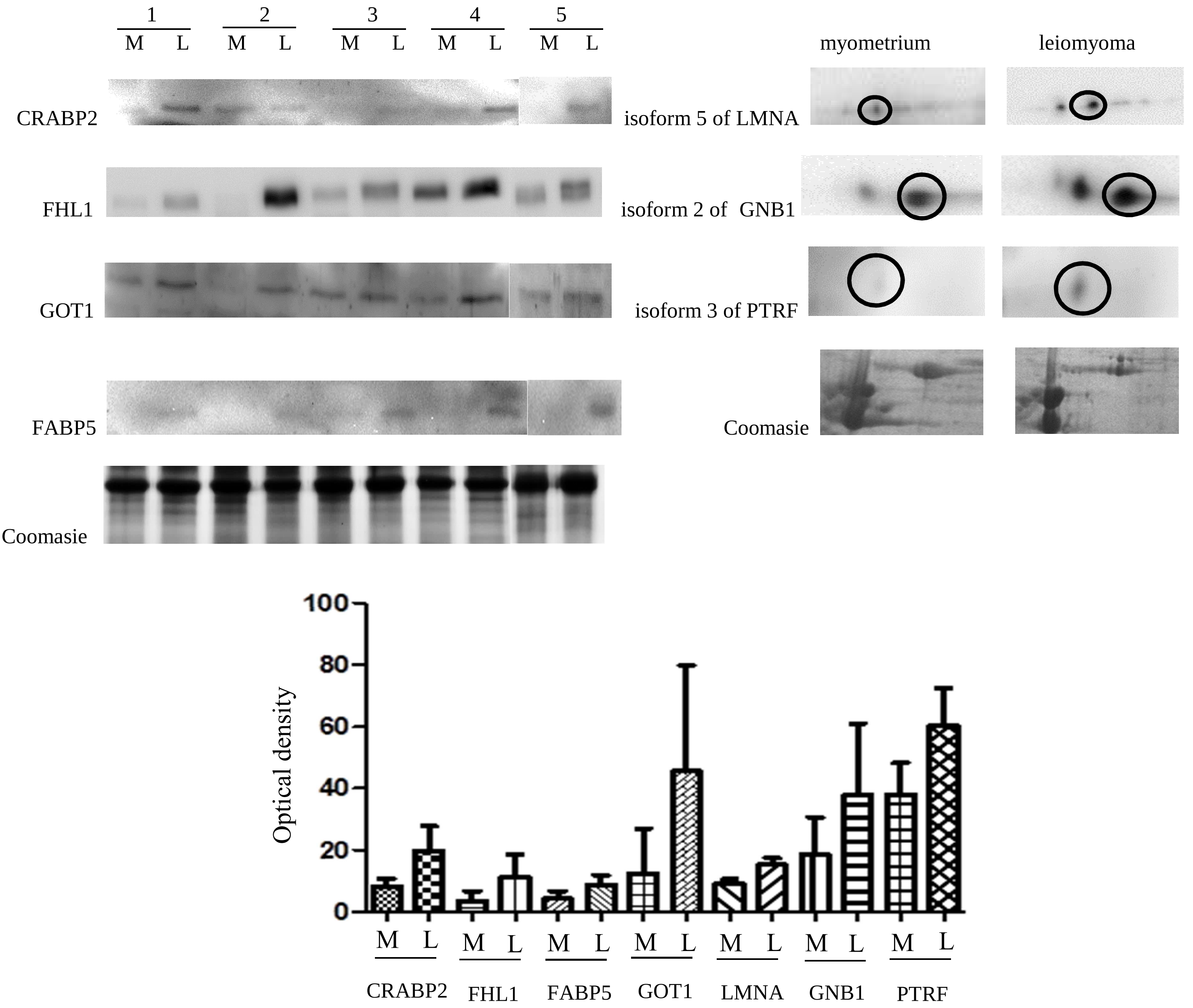

Figure S3. Western blot analysis of CRABP2, FHL1, FABP5, GOT1, isoform 5 of LMNA, isoform 2 of GNB1, isoform 3 of PTRF in paired myometrium (M) and leiomyoma (L). The isoform corresponding to the one identified by 2-DE was circled. The intensity of immunostained bands was normalized against the total protein intensities measured from the same blot stained with Coomassie brilliant blue. The bar graph shows the relative expression (band density) of proteins in the myometrium and the leiomyoma. Results are shown as a histogram ($p < 0.05$) and each bar represents mean ± SD.
