# Supplementary material for: A Proteomic Approach for the Identification of Up-Regulated Proteins Involved in the Metabolic Process of the Leiomyoma
Source: Int J Mol Sci. 2016 Apr 9;17(4):540. doi: 10.3390/ijms17040540 (PMC4848996; doi:10.3390/ijms17040540)
Supplement: Supplementary file 1 [file ijms-17-00540-s001.zip › ijms-109333-supplementary-revise 1/Table S1.pdf]

SUPPLEMENTARY TABLE 1. Spots and list of peptides identified by MALDI-TOF/TOF

Spot n	Protein description	Molecular weight	Isoelectric point	Peptides
1	Protein disulfide isomerase A3	54.9	6.86	FLQDYFDGNLK
				TFSHELSDFGLESTAGE IPVVAIR
6	Myosin regulatory light polypeptide 9	19.8	4.92	LNGTDPEDVIR
				FTDEEVDEmYR
7	Desmin	53.4	5.27	FASEASGYQDNIAR
				INLPIQTYSALNFR
12	Four and a half LIM domains protein 1	24.7	8.6	FDCHYCR
				FWHDTCFR
				QVIGTGSFFPK
				CLHPLANETFVAK
13	Keratin, type II cytoskeletal 1	66.2	8.15	LALDLEIATYR
				SLNNQFASFIDKVR
20	Immunoglobulin heavy constant alpha 1	38.5	6.08	WLQGSQELPR
				QEPSQGTTTFAVTSILR
22	isoform 5 of Prelamin-A/C	63.1	6.84	SSFSQHAR
				NIYSEELR
				NIYSEELR
				LADALQELR
				TQSPQNCSIM
				TQSPQNCSIM
				TLEGELHDLR
				LRDLEDSLAR
				AQNTWGCGNSLR
				NIYSEELRETK
				TALINSTGEEVAMR
				AQHEDQVEQYKK
				VAVEEVDEEGKFVR
				VAVEEVDEEGKFVR
				TLEGELHDLRGQVAK
				IRIDSLSAQLSQLQK
				IRIDSLSAQLSQLQK
				NSNLVGAAHEELQQSR
				NSNLVGAAHEELQQSR
				AGQVVTIWAAGAGATHSPPTDLVWK
16	AP-1 complex subunit mu-2	48.1	8.23	RDPAMLPK
				SASAVFILDVK
				MSASAVFILDVK
				SASAVFILDVKGK
15	LIM and SH3 domain protein 1 fragment	19.0	9.04	GFSVVADTPELQR
				LKQQSELQSQVR
				LKQQSELQSQVR
17	T-complex protein 1 subunit epsilon	57.6	5.37	NLIRDNR
				NLIRDNR

				NSSLGPTIEK
				GGNKMIIEEAK
				MLVIEQCKNSR
				DVDFELIKVEGK
				QMAEIAVNAVLTVADMERR
				DGDVTVTNDGATILSMMDVDHQIAK
19	Malate dehydrogenase cytoplasmic	23.2	6.9	GEFVTTVQQR
				GEFVTTVQQR
				FVEGLPINDFSR
				SAPSIPKENFSCLTR
				LSSAMSAAKAICDHVR
				MVIVVGNPANTNCLTASK
23	Actin, alpha cardiac muscle 1	42.3	5.23	AGFAGDDAPR
				GYSFVTTAER
				AVFPSIVGRPR
				QEYDEAGPSIVHR
				IWHHTFYNELR
24	Alpha-1-antitrypsin	46.7	5.47	DTEEEDFHVDQVTTVK
				VFSNGADLSGVTEEAPLK
				ITPNLAEFAFSLYR
				TDTSHHDQDHPTFNK
				SVLGQLGITK
				LSITGTYDLK
21	Serum albumin	53.0	6.62	YLYEIAR
				RHPDYSVVLLLR
