# Supplementary material for: A Proteomic Approach for the Identification of Up-Regulated Proteins Involved in the Metabolic Process of the Leiomyoma
Source: Int J Mol Sci. 2016 Apr 9;17(4):540. doi: 10.3390/ijms17040540 (PMC4848996; doi:10.3390/ijms17040540)
Supplement: Supplementary file 1 [file ijms-17-00540-s001.zip › ijms-109333-supplementary-revise 1/Table S2.pdf]

SUPPLEMENTARY TABLE 2. Spots and list of peptides identified by LTQ-Orbitrap XL

Spot n	Protein description	Molecular weight	Isoelectric point	Peptides
2	Tubulin beta chain	47.7	4.81	TAVcDIPPR
				EVDEQmLNVQNK
				ISVYYNEATGGK
				ImNTFSVVPSPK
				ISEQFTAmFR
3	L-lactate dehydrogenase B chain	36.6	6.05	VIGSGcNLDSAR
				IVVVTAGVR
				IVADKDYSVTANSK
				SADTLWDIQK
				LKDDEVAQLK
				LIAPVAEEEATVPNNK
				GLTSVINQK
				mVVESAYEVIK
				DYSVTANSK
				LNLVQR
				KSADTLWDIQK
				MVVESAYEVIK
				NVNVFK
				YLmAEK
				LSGLPK
4	Annexin A4	35.5	5.86	AASGFNAmEDAQTLR
				GLGTDDNTLIR
				QDAQDLYEAGEKK
				DEGNYLDDALVR
				AEIDmLDIR
				VLVSLSAGGR
				QDAQDLYEAGEK
				VLLVLcGGDD
				SDTSFmFQR
				ISQTYQQQYGR
				GLGTDEDAIISVLAYR
				AASGFNAmEDAQTLRK
				SDTSFMFQR
				AEIDMLDIR
				SAYFAEK
				FLTVLcSR
				TPEEIR
				DIEQSIK
				SLEDDIR
				RISQTYQQQYGR
5	isoform 2 of Guanine nucleotide-binding protein G(I)/G(S)/G(T) subunit beta	36.3	6	LLVSASQDGK
				LFVSGAcDASAK
				AGVLAGHDNR
10	Cellular retinoic acid-binding protein 2	15.7	5.4	PNFSGNWK
				VGEEFEEQTVDGRPcK
				LLKGEGPK
				IAVAAASKPAVEIK

				VLGVNVmLR
				QEGDTFYIK
				VYVRE
8	Calmodulin 1	16.8	4.22	DTDSEEEIR
				mKDTDSEEEIR
				ELGTVmR
9	isoform3 of Polymerase I and transcript release factor	22.6	7.56	EGQVEVLK
				ATEmVEVGADDDEGGAER
				ATEMVEVGADDDEGGAER
11	Fatty acid binding protein, epidermal	15.2	7.01	FEETTADGR
				LVVEcVmNNVTcTR
				ELGVGIALR
				TTQFScTLGEK
				GFDEYmK
				FEETTADGRK
				ATVQQLEGR
18	Aspartate aminotransferase, cytoplasmic	46.2	7.01	LALGDDSPALK
				VGGVQSLGGTGALR
				IGADFLAR
				LALGDDSPALK
				INVSGLTTK
				VGGVQSLGGTGALR
				LALGDDSPALKEK
				ITWSNPPAQGAR
				INVSGLTTK
				ITWSNPPAQGAR
				VNLGVGAYR
				VGGVQSLGGTGALR
				NLDYVATSIHEAVTK
14	Keratin, type I cytoskeletal 9	62.0	5.24	SGGGGGGGLGSGGSIR
				SGGGGGGGLGSGGSIR
				GGSGGSYGGGGSGGGYGGGSGSR
				SGGGGGGGLGSGGSIR
				GGSGGSHGGGSGFGGESGGSYGGGEEASGSGGGYGGGSGK
				GGSGGSYGGGGSGGGYGGGSGSR
				FSSSSGYGGGSSR
				GGSGGSHGGGSGFGGESGGSYGGGEEASGSGGGYGGGSGK
				GGSGGSYGGGGSGGG YGGGSGSR
