# Supplementary figures and images for: A Proteomic Approach for the Identification of Up-Regulated Proteins Involved in the Metabolic Process of the Leiomyoma
Source: Int J Mol Sci. 2016 Apr 9;17(4):540. doi: 10.3390/ijms17040540 (PMC4848996; doi:10.3390/ijms17040540)

Figure S2: Visualization of spots for down-regulated proteins

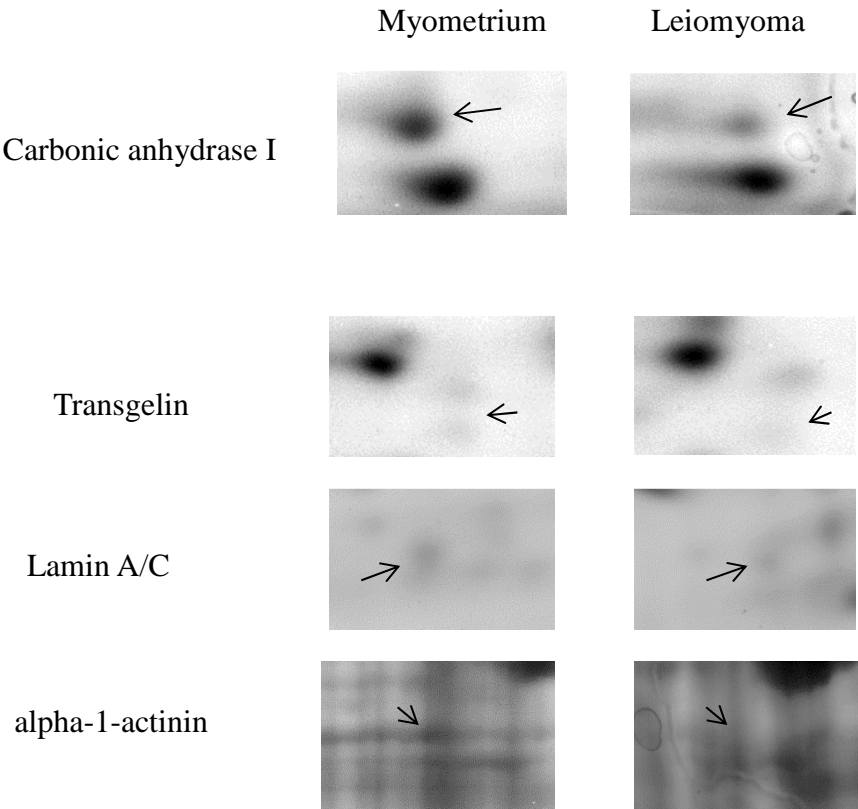

Supplement: Supplementary file 1 [file ijms-17-00540-s001.zip › ijms-109333-supplementary-revise 1/Figure S2.pdf]
